# Safety and efficacy of intravenous hydromorphone patient-controlled analgesia versus intramuscular pethidine in acute pancreatitis: An open-label, randomized controlled trial

**DOI:** 10.3389/fphar.2022.962671

**Published:** 2022-08-04

**Authors:** Zhiyao Chen, Kun Jiang, Fei Liu, Ping Zhu, Fei Cai, Yanqiu He, Tao Jin, Ziqi Lin, Qian Li, Cheng Hu, Qingyuan Tan, Xiaonan Yang, Jia Guo, Wei Huang, Lihui Deng, Qing Xia

**Affiliations:** ^1^ Pancreatitis Center, Center of Integrated Traditional Chinese and Western Medicine, West China Hospital, Sichuan University, Chengdu, China; ^2^ Department of Anesthesiology, West China Hospital, Sichuan University, Chengdu, China

**Keywords:** acute pancreatitis, hydromorphone, pethidine, patient-controlled analgesia, randomized controlled trial

## Abstract

**Background:** Hydromorphone patient-controlled analgesia (PCA) provides satisfactory postoperative pain therapy, but its effect has not been assessed in acute pancreatitis (AP).

**Aim:** To assess the safety and efficacy of intravenous hydromorphone PCA for pain relief in AP.

**Methods:** This open-label trial included AP patients admitted within 72 h of symptom onset, aged 18–70 years old, and with Visual Analog Scale (VAS) for pain intensity ≥5. They were randomized to receive intravenous hydromorphone PCA (0.05 mg/h with 0.2 mg on-demand) or intramuscular pethidine (50 mg as required) for three consecutive days. Intramuscular dezocine (5 mg on demand) was the rescue analgesia. The primary outcome was the change of VAS score recorded every 4 h for 3 days. Interim analysis was conducted by an Independent Data and Safety Monitoring Committee (IDSMC).

**Results:** From 26 July 2019 to 15 January 2020, 77 patients were eligible for the intention-to-treat analysis in the interim analysis (39 in the hydromorphone group and 38 in the pethidine group). Baseline parameters were comparable between groups. No difference in VAS between the two groups was found. Hydromorphone PCA was associated with higher moderately severe to severe cases (82.1% vs. 55.3%, *p* = 0.011), acute peripancreatic fluid collections (53.9% vs. 28.9%, *p* = 0.027), more cumulative opioid consumption (median 46.7 vs. 5 mg, *p* < 0.001), higher analgesia costs (median 85.5 vs. 0.5 $, *p* < 0.001) and hospitalization costs (median 3,778 vs. 2,273 $, *p* = 0.007), and more adverse events (20.5% vs. 2.6%, *p* = 0.087). The per-protocol analysis did not change the results. Although a sample size of 122 patients was planned, the IDSMC halted further recruitment as disease worsening or worse clinical outcomes between the groups in the interim analysis.

**Conclusion:** Hydromorphone PCA was not superior to pethidine in relieving pain in AP patients and might have worse clinical outcomes. Therefore, its use is not recommended.

**Clinical Trial Registration:** Chictr.org.cn. ChiCTR1900025971

## 1 Introduction

Acute pancreatitis (AP) is a critical digestive disease, with severe pain as one of its cardinal symptoms, often necessitating analgesia (Drewes et al., 2020). Pain is included as the fifth vital sign and one of the diagnostic criteria for AP (Banks et al., 2013; Morone and Weiner, 2013). Although no solid evidence shows that the intensity of pain correlates with disease severity (Kapoor et al., 2013), the importance of abdominal pain has been considered in the Pancreatitis Activity Scoring System (PASS) in 2017 (Wu et al., 2017), which was correlated with AP clinical outcomes (Buxbaum et al., 2018; Thiruvengadam et al., 2021). Therefore, analgesia is a clinical priority for AP management.

Parenteral analgesics, such as opioids, nonsteroidal anti-inflammatory drugs (NSAIDs), and other adjuvant analgesics, are suggested for pain relief of AP (Basurto Ona et al., 2013; Moggia et al., 2017). Opioids, strong pain killers used in 93% of AP patients in North America (Matta et al., 2020), have been evaluated in over 70% of AP randomized controlled trials (RCTs) about analgesics for pain relief (Blamey et al., 1984; Ebbehøj et al., 1985; Jakobs et al., 2000; Stevens et al., 2002; Kahl et al., 2004; Peiró et al., 2008; Layer et al., 2011; Sadowski et al., 2015; Gülen et al., 2016; Mahapatra et al., 2019; Huang et al., 2020; Kumar et al., 2020). The first RCT on opioid use, with 32 AP participants, showed no difference between intramuscular buprenorphine and intramuscular pethidine for pain relief (Blamey et al., 1984). Another trial showed that epidural analgesia of a combination of bupivacaine and fentanyl increased arterial perfusion of the pancreas to a higher degree than that in fentanyl in patient-controlled analgesia (PCA) (Sadowski et al., 2015), and was the only study reporting the use of PCA in AP patients. One recent RCT showed that the opioid pentazocine had better efficacy than the NSAID diclofenac in AP (Mahapatra et al., 2019). In contrast, another trial concluded that diclofenac and tramadol were equally effective in AP pain management (Kumar et al., 2020). However, these studies had relatively small sample sizes, included participants with mild acute pancreatitis (MAP), and adopted different diagnostic criteria for AP. In our recent systematic review and meta-analysis, NSAIDs and opioids are equally effective for analgesia in MAP, but the optimal analgesic strategy for moderately severe acute pancreatitis (MSAP) and severe acute pancreatitis (SAP) patients remains unclear (Cai et al., 2021).

The use of opioids should be individualized, following a gradual addition of small doses, and an ordinary intramuscular injection or intravenous infusion cannot achieve this purpose. Patients frequently request the use of painkillers due to unbearable pain, which increases the workload of the medical staff and reduces the efficiency of their work. PCA achieves satisfactory analgesia by allowing patients to control their medication doses. A systematic review showed that opioid PCA provided better pain control in postoperative patients (McNicol et al., 2015). Hydromorphone, a semi-synthetic opioid agonist clinically applied since 1926 (Murray and Hagen, 2005), plays its analgesic role by stimulating the central nervous system μ-opioid receptors and is widely used for acute, chronic and cancerous pains (Quigley and Wiffen, 2003; Bao et al., 2016). A meta-analysis of eight studies showed that hydromorphone had better analgesic effects than morphine (Felden et al., 2011). However, this notion has never been tested in the AP setting. Although the 2019 World Society of Emergency Surgery (WSES) recommended the use of PCA and hydromorphone in AP due to their superiority to morphine (Leppäniemi et al., 2019), most AP guidelines lack a recommendation regarding optimal pain medications and analgesic approaches (Cai et al., 2021).

In this study, we aimed to evaluate the safety and efficacy of intravenous hydromorphone PCA for pain relief in AP. Based on better analgesic results provided by previous studies comparing PCA with conventional intramuscular pethidine (Searle et al., 1994; Wilson et al., 2018), we hypothesized that intravenous hydromorphone PCA would achieve a better effect of pain relief than intramuscular pethidine in AP patients.

## 2 Materials and methods

### 2.1 Study design and registration

We carried out an open-label RCT in the Department of Integrated Traditional Chinese and Western Medicine, West China Hospital of Sichuan University. Consolidated Standards of Reporting Trials (CONSORT) guidelines were used to design this trial (Schulz et al., 2010). Before trial implementation, we obtained approval from the Ethics Committee on Biomedical Research, West China Hospital of Sichuan University (Number 2019511) and completed the clinical trial registration on the Chinese Clinical Trial Registry website (Number ChiCTR1900025971).

### 2.2 Eligibility criteria for participants

All patients were considered eligible if they met the following inclusion criteria: 1) a definite diagnosis of AP by the revised Atlanta classification (Banks et al., 2013); 2) aged from 18 to 70 years; 3) admission within 72 h from abdominal pain onset; and 4) the Visual Analog Scale (VAS) score at admission was greater than or equal to five scores (Jensen et al., 1986). Ineligible patients were excluded if they met the exclusion criteria: 1) known pregnant or lactating at admission; 2) patients with acute onset of chronic pancreatitis, acute traumatic pancreatitis and recurrent acute pancreatitis; 3) patients with severe chronic diseases such as coronary heart disease, chronic obstructive pulmonary disease, liver and kidney dysfunction, anemia, mental illness and malignant tumors; 4) patients suffering from contraindications to hydromorphone or pethidine such as severe pulmonary insufficiency, paralytic ileus, supraventricular tachycardia, traumatic brain injury, and intracranial space-occupying lesions; 5) patients who were allergic to hydromorphone or pethidine; 6) patients unwilling to sign the informed consent form.

### 2.3 Termination criteria

Interim analysis was conducted by an Independent Data and Safety Monitoring Committee (IDSMC), which also determined whether the trial should be halted. The termination criteria were as follows: 1) patients who suffered severe adverse events (SAEs) related to the intervention drugs; 2) the planned interim analysis achieved the expected outcome differences; and 3) interim analysis showed disease worsening or worse clinical outcomes between the groups.

### 2.4 Management of drop-out cases

The definition of a drop-out case was any patient who was enrolled in the trial but quit the study for any reason. If a patient dropped out, a researcher completed a case report form, outlining their reasons. For patients who dropped out due to adverse events (AEs), researchers closely followed up on their conditions until AEs disappeared. All the drop-out cases could not be replaced and were included in the intention-to-treat analysis.

### 2.5 Randomization and masking

After the inclusion and exclusion criteria were screened, eligible patients were randomized in a 1:1 ratio to receive intravenous hydromorphone PCA or intramuscular pethidine. An independent researcher generated random numbers using SPSS (Version 21, IBM, Armonk, New York, United States ) before the first subject was recruited. Random numbers were kept in a sealed envelope in the order of their selection. An independent hospital staff member who was available 24 h, 7 days a week by telephone kept the sealed envelopes to ensure the concealment of the allocation sequence. Due to the different patterns of the two drug administrations, the study participants and researchers were not blinded to the study group assignment.

### 2.6 Intervention

#### 2.6.1 Acute pancreatitis treatment

All participants received the same treatment (Tenner et al., 2013; Crockett et al., 2018), including fluid resuscitation, nutrition support, organ function support, antibiotics with indications, and surgical intervention, if necessary.

#### 2.6.2 Administration of study medications

In the hydromorphone group, 10 mg of hydromorphone (2 ml: 2 mg) was mixed with 0.9% saline to a volume dose of 200 ml. The PCA pump (Rehn MedTech Co. Ltd. Nantong, Jiangsu, China) was programmed by a specialized anesthetist using background infusion at 0.05 mg every hour, a demand dose of 0.2 mg each time. Patients were trained to press the button on the PCA pump when they felt pain. To avoid hydromorphone overdosage, the pump was automatically stopped from transfusing hydromorphone with a lockout period of 10 min, and a 1-h maximum dose of 1.2 mg. A PCA pump was used for three consecutive days in the hydromorphone group. In the pethidine group, patients were given intramuscular pethidine (50 mg) on demand for three consecutive days.

#### 2.6.3 Rescue medication

If patients still complained of insufferable pain after PCA or pethidine administration after 3 days, intramuscular dezocine (5 mg) was used as the rescue analgesic.

#### 2.6.4 Forbidden medications

During hospitalization, the routine use of the following therapeutics was not allowed: 1) acupuncture; 2) ultrasonic analgesic therapy; and 3) other analgesics, such as NSAIDs and opioid analgesics.

### 2.7 Outcome measures

The primary outcome was a change in the VAS score, which was recorded every 4 h for 3 days. During the time points corresponding to nighttime, when patients were sleeping, VAS was recorded as SLEEP, and the scores were recorded as zero (Wu et al., 2017). Secondary outcomes included: 1) daily evaluation of clinical scores in the first 3 days after admission, including the Modified Marshall score (Marshall et al., 1995), Sequential Organ Failure Assessment (SOFA) score (Ferreira et al., 2001), Bedside index of severity in acute pancreatitis (BISAP) score (Wu et al., 2008), Acute physiology and chronic health evaluation (APACHE II) (Larvin and McMahon, 1989), and PASS; 2) serum C-reactive protein (CRP), tumor necrosis factor (TNF)-α, procalcitonin (PCT), interleukin (IL)-6, on admission and on the 4th day after admission; 3) organ failure (OF) occurrence; 4) local complications, such as acute peripancreatic fluid collection (APFC) and acute necrotic collection (ANC) as per revised Atlanta criteria, which were evaluated by contrast-enhanced computed tomography (CECT); 5) in-hospital mortality; 6) opioid consumption was calculated based on equivalent morphine doses (Mcpherson, 2018); 7) length of hospital stay; and 8) costs of analgesics and hospitalization.

### 2.8 Adverse events and safety assessment

AEs and SAEs were defined in accordance with the National Cancer Institute Common Terminology Criteria for Adverse Events v4.0. Common side effects of the study medication were closely monitored and recorded (Els et al., 2017; Verberkt et al., 2017). The time window for AEs was defined as those occurring within 72 h after PCA or within 2 h after pethidine injection.

### 2.9 Sample size calculation and statistical analysis

In a previous study (Blamey et al., 1984), the linear analog scale of pain after AP patients received intramuscular pethidine was 3.6. In this study, we anticipated that the patient’s VAS score after receiving hydromorphone would be 2. The standard deviation (SD) in the hydromorphone and pethidine groups was 3. Assuming a significance level of 0.05 and a study power of 0.8 with a 10% drop-out rate, 122 patients were required for this trial, with 61 patients in each group. The sample size was calculated by Power Analysis and Sample Size (Version 11.0.7, NCSS).

Statistical analyses were performed using SPSS 21. Continuous data were expressed as medians and interquartile ranges. Categorical data were presented as numbers and percentages. Continuous variables were compared using the independent-sample *t* test or the Mann–Whitney *U* test (for non-normal distributions). Categorical variables were compared with the Chi-squared test or Fisher’s exact test (for 2 × 2 tables with cells under 5). Two-sided *p*-values less than 0.05 indicated statistically significant differences. Baseline characteristics and clinical outcomes were described based on the intention-to-treat population, which included participants who had at least one treatment and one primary outcome measure (*n* = 77). Per-protocol analysis was performed to test the efficacy of treatment measures, which included participants completing the treatment plan as per the protocol (*n* = 72). Figures were performed using GraphPad Prism (Version 8, San Diego, California, United States ).

## 3 Results

After the interim analysis of the inclusion of 77 participants from 26 July 2019 to 15 January 2020, the IDSMC suggested the termination of participant recruitment, as increased MSAP to SAP and a higher incidence of APFC occurred in the hydromorphone group versus the pethidine group, greatly threatening participant health.

### 3.1 Participants and baseline characteristics

**FIGURE 1:**
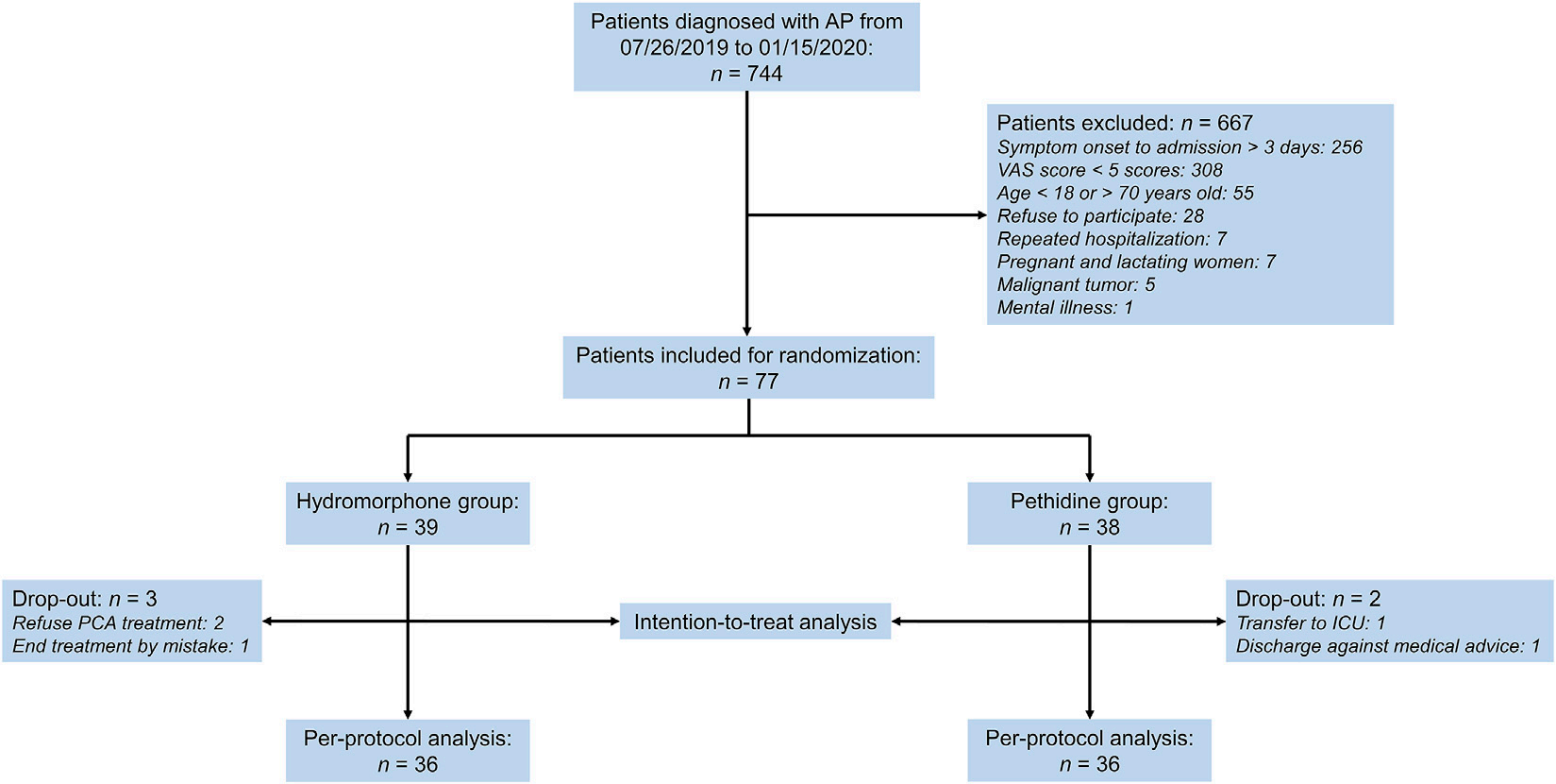
Patient selection flowchart. AP, acute pancreatitis, VAS, visual analog scale score, PCA, patient-controlled analgesia, ICU, intensive care unit.

In the initial screening of 744 AP patients, 77 participants were randomized (39 in the hydromorphone group and 38 in the pethidine group) and included in the intention-to-treat population. Five patients (three in the hydromorphone group and two in the pethidine group) dropped out. Among them, two refused to use PCA halfway, one had their PCA pump taken away by mistake within the first 72 h after enrollment, one patient received sedation treatment in the intensive care unit (ICU) and could not control the pump, and one patient abandoned treatment and had to be discharged. The patient selection process is shown in Figure 1.

In total of 77 patients, there were 54 males and 23 females, and the mean age was 44.9 years. Hypertriglyceridemia (37.7%) and biliary (31.2%) were the main etiologies, consistent with our previous studies (Zhang et al., 2019; Li et al., 2020; Shi et al., 2020). Of the 29 cases of hypertriglyceridemia-induced AP, only one patient had a history of alcohol abuse in the hydromorphone group. The baseline characteristics of the included patients are provided in Table 1. The gender distribution, age, etiology, intervals from onset to admission, Charlson comorbidity index, and clinical severity scores of participants were similar (*p* > 0.05). The respiratory rate in the hydromorphone group was higher than that in the pethidine group (22, IQR 20–26 versus 20, IQR 20–22, *p* = 0.036). There were no significant differences in the serum levels of triglycerides, amylase, lipase, urea and creatinine between the two groups (*p* > 0.05).

**TABLE 1:** Baseline characteristics of patients based on intention-to-treat and per-protocol analyses.

Parameters	Intention-to-treat analysis			Per-protocol analysis		
Group	Hydromorphone (*n* = 39)	Pethidine (*n* = 38)	*p*-value	Hydromorphone (*n* = 36)	Pethidine (*n* = 36)	*p*-value
Gender (m/f)	27/12	27/11	0.861	24/12	27/9	0.437
Age (y, mean, s.d.)	43.5 (10.2)	46.3 (10.4)	0.241	43.4 (10.3)	45.8 (10.6)	0.329
BMI (kg/m^2^, mean, s.d.)	26.1 (3.2)	25.1 (3.1)	0.180	25.9 (3.2)	25.0 (3.0)	0.247
Etiology, n (%)			0.871			0.756
Hypertriglyceridemia	15 (38.5)	14 (36.8)		15 (41.6)	13 (36.1)	
Biliary	12 (30.8)	12 (31.6)		12 (33.3)	11 (30.6)	
Alcohol	4 (10.3)	6 (15.8)		3 (8.3)	6 (16.7)	
Others	8 (20.5)	6 (15.8)		6 (16.7)	6 (16.7)	
Time from onset to admission (h), median (IQR)	20 (10–42)	21 (12–30)	0.899	22 (9–42)	23 (12–35)	0.960
Charlson comorbidity index, (median) (IQR)	1 (0–1)	1 (1–2)	0.252	1 (0–1)	1 (1–1.8)	0.315
Heart rate, mean (s.d.)	101 (21)	95 (23)	0.253	101 (21)	94 (23)	0.182
Respiratory rate, median (IQR)	22 (20–26)	20 (20–22)	**0.036**	22 (20–26)	20 (20–22)	**0.030**
Clinical scores, median (IQR)						
Modified Marshall	1 (1–2)	1 (1–2)	0.364	1 (0–2)	1 (1–2)	0.645
SOFA	2 (1–4)	2 (1–3)	0.163	2 (1–4)	2 (1–3)	0.224
APACHE II	6 (4–11)	5 (4–7)	0.301	6 (4–11)	5 (4–7)	0.295
BISAP	1 (1–2)	1 (0–1)	0.472	1 (0–2)	1 (0–1)	0.399
HAPS	1 (1–2)	2 (1–2)	0.266	1 (1–2)	2 (1–2)	0.219
MCTSI	4 (4–6)	4 (2–6)	0.258	4 (4–6)	4 (2–6)	0.180
CTSI	2 (2–4)	2 (2–4)	0.751	2 (2–4)	2 (2–4)	0.529
EPIC	1 (0–3)	2 (0–3)	0.592	1 (0–3)	2 (0–3)	0.584
PASS	140 (115–175)	123 (94–158)	0.244	140 (116–175)	120 (91–163)	0.175
Laboratory makers, median (IQR)						
Triglycerides (mmol/L)	10.46 (1.4–13.94)	8.98 (1.48–14.49)	0.756	10.96 (1.12–14.26)	8.98 (1.57–14.33)	0.809
Urea (mmol/L)	4.8 (3.6–6.4)	4.6 (3.7–6.1)	0.927	4.65 (3.45–6.70)	4.35 (3.55–6.08)	0.848
Creatinine (umol/L)	70 (59–83)	64 (56–85)	0.756	69 (59–82)	64 (57–86)	0.870
Amylase (IU/L)	575 (245–901)	334 (120–737)	0.175	583 (265–977)	334 (104–895)	0.136
Lipase (IU/L)	795 (428–1,450)	567 (180–1,312)	0.243	800 (436–1,450)	546 (157–1,319)	0.180
CRP (mg/L)	172 (23–318)	118 (12–249)	0.256	171 (25–314)	121.5 (16–254)	0.368

IQR, interquartile range; BMI, body mass index; SOFA, sequential organ failure assessment; APACHE II, acute physiology and chronic health evaluation; BISAP, bedside index of severity in acute pancreatitis; HAPS, the harmless acute pancreatitis score; MCTSI, modified computed tomography severity index; CTSI, computed tomography severity index; EPIC, extra-pancreatic inflammation on computed tomography score; PASS, pancreatitis activity scoring system; CRP, C-reactive protein.

Statistical significance between groups (*p* ≤ 0.05) is indicated in bold.

### 3.2 Primary outcome

**FIGURE 2:**
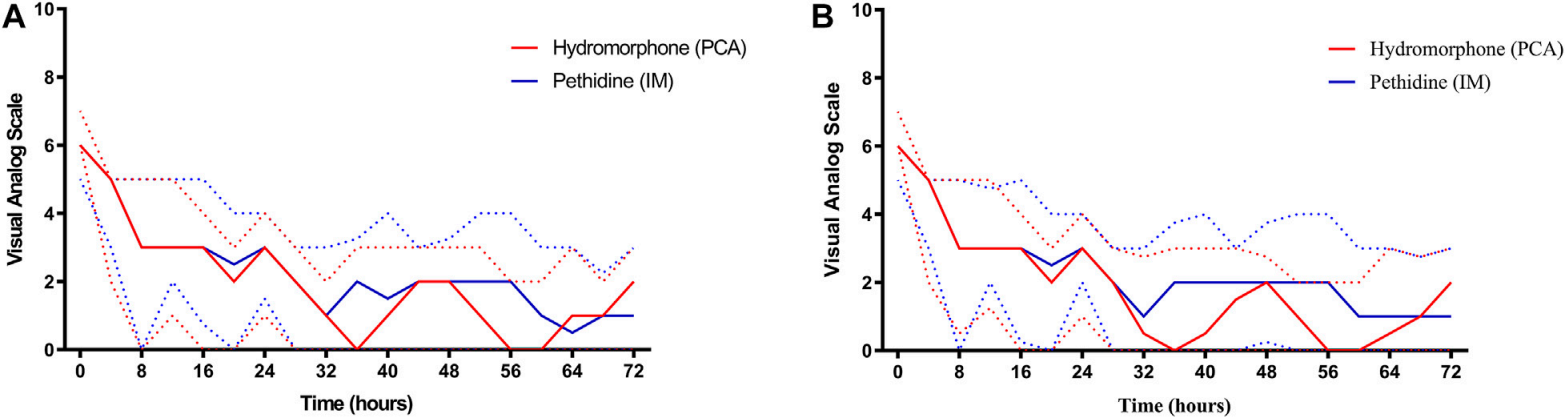
Trends of VAS scores in the two groups. Solid red and solid blue lines represent changes in VAS score over 72 h in the hydromorphone and pethidine groups, respectively. Dotted red and dotted blue lines represent the interquartile range of VAS scores in the hydromorphone and pethidine groups, respectively. **(A)** VAS score based on intention-to-treat analysis. **(B)** VAS score based on per-protocol analysis. PCA, patient-controlled analgesia, IM, intramuscular, VAS, visual analog scale score.

On admission, the median VAS scores of both groups were similar (6, IQR 6–7 in the hydromorphone group versus 6, IQR 5–6 in the pethidine group, *p* = 0.261) (Figure 2). Thereafter, the VAS scores of both groups declined. The lowest VAS score in the hydromorphone group was 0 and was reported during the 36th hour, while that in the pethidine group was 0.5 and was detected during the 64th hour. There were no significant differences at any timepoints between the groups (*p* > 0.05) (Table 2). Moreover, the cumulative VAS scores for the first 24 h, 24–48 h, and 48–72 h showed no significant differences (*p* > 0.05) (Supplementary Table S1).

**TABLE 2:** VAS score trends over 72 h based on intention-to-treat and per-protocol analyses.

Parameters	Intention-to-treat analysis			Per-protocol analysis		
Group	Hydromorphone (*n* = 39)	Pethidine (*n* = 38)	*p*-value	Hydromorphone (*n* = 36)	Pethidine (*n* = 36)	*p*-value
0 h VAS, median (IQR)	6 (6–7)	6 (5–6)	0.261	6 (6–7)	6 (5–6)	0.246
4 h VAS, median (IQR)	5 (2–5)	5 (3–5)	0.513	5 (2–5)	5 (3–5)	0.615
8 h VAS, median (IQR)	3 (0–5)	3 (0–5)	0.795	3 (0.5–5)	3 (0–5)	0.805
12 h VAS, median (IQR)	3 (1–5)	3 (2–5)	0.852	3 (1.25–5)	3 (2–4.75)	0.651
16 h VAS, median (IQR)	3 (0–4)	3 (0.75–5)	0.369	3 (0–4)	3 (0.25–5)	0.539
20 h VAS, median (IQR)	2 (0–3)	2.5 (0–4)	0.564	2 (0–3)	2.5 (0–4)	0.475
24 h VAS, median (IQR)	3 (1–4)	3 (1.5–4)	0.803	3 (1–4)	3 (2–4)	0.687
28 h VAS, median (IQR)	2 (0–3)	2 (0–3)	0.587	2 (0–3)	2 (0–3)	0.522
32 h VAS, median (IQR)	1 (0–2)	1 (0–3)	0.935	0.5 (0–2.75)	1 (0–3)	0.760
36 h VAS, median (IQR)	0 (0–3)	2 (0–3.25)	0.112	0 (0–3)	2 (0–3.75)	0.099
40 h VAS, median (IQR)	1 (0–3)	1.5 (0–4)	0.350	0.5 (0–3)	2 (0–4)	0.225
44 h VAS, median (IQR)	2 (0–3)	2 (0–3)	0.954	1.5 (0–3)	2 (0–3)	0.700
48 h VAS, median (IQR)	2 (0–3)	2 (0–3.25)	0.524	2 (0–2.75)	2 (0.25–3.75)	0.228
52 h VAS, median (IQR)	1 (0–3)	2 (0–4)	0.310	1 (0–2)	2 (0–4)	0.109
56 h VAS, median (IQR)	0 (0–2)	2 (0–4)	0.168	0 (0–2)	2 (0–4)	0.101
60 h VAS, median (IQR)	0 (0–2)	1 (0–3)	0.608	0 (0–2)	1 (0–3)	0.449
64 h VAS, median (IQR)	1 (0–3)	0.5 (0–3)	0.595	0.5 (0–3)	1 (0–3)	0.986
68 h VAS, median (IQR)	1 (0–2)	1 (0–2.25)	0.979	1 (0–2.75)	1 (0–2.75)	0.813
72 h VAS, median (IQR)	2 (0–3)	1 (0–3)	0.459	2 (0–3)	1 (0–3)	0.825

IQR, interquartile range; VAS, visual analog scale score.

### 3.3 Secondary outcomes

The incidence of MSAP to SAP (82.1% versus 55.3%, *p* = 0.011) and APFC (53.9% versus 28.9%, *p* = 0.027) was higher in the hydromorphone group than those in the pethidine group (Table 3). The two groups had no significant difference in organ failure, persistent organ failure, ICU admission, length of hospitalization, and mortality (*p* > 0.05).

**TABLE 3:** Secondary outcomes of the two groups based on intention-to-treat and per-protocol analyses.

Parameters	Intention-to-treat analysis			Per-protocol analysis		
Group	Hydromorphone (*n* = 39)	Pethidine (*n* = 38)	*p*-value	Hydromorphone (*n* = 36)	Pethidine (*n* = 36)	*p*-value
Organ failure, n (%)						
Transient organ failure	13 (33.3)	6 (15.8)	0.074	10 (27.8)	6 (16.7)	0.260
Persistent organ failure	12 (30.8)	9 (23.7)	0.485	12 (33.3)	7 (19.4)	0.181
Persistent organ failure, n (%)						
Respiratory	12 (30.8)	9 (23.7)	0.485	12 (33.3)	7 (19.4)	0.181
Cardiovascular	1 (2.6)	1 (2.6)	1.000	0	1 (2.8)	1.000
Renal	0	1 (2.6)	0.990	0	0	—
HDU/ICU admission, n (%)	20 (51.3)	13 (34.2)	0.130	19 (52.8)	11 (30.6)	0.056
Local complication, n (%)	24 (61.5)	15 (39.5)	0.053	22 (61.1)	14 (38.9)	0.059
APFC	21 (53.9)	11 (28.9)	**0.027**	19 (52.8)	8 (22.2)	**0.008**
ANC	11 (28.2)	6 (15.8)	0.189	10 (27.8)	4 (11.1)	0.076
Severity, n (%)			**0.037**			**0.042**
Mild	7 (17.9)	17 (44.7)		7 (19.4)	17 (47.2)	
Moderately Severe	20 (51.3)	12 (31.6)		17 (47.2)	12 (33.3)	
Severe	12 (30.8)	9 (23.7)		12 (33.3)	7 (19.4)	
Mortality, n (%)	1 (2.6)	1 (2.6)	0.985	1 (2.8)	0	0.317
Hospital staying (d), median (IQR)	11 (8–15)	9 (7–14)	0.136	11 (8–15)	9 (7–14)	0.122
Cumulative opioid consumption (mg), median (IQR)	46.7 (31.5–67.2)	5 (0–10)	**< 0.001**	50.3 (33.2–68.5)	5 (0–10)	**< 0.001**
Total dose of dezocine needed as the rescue analgesic (mg), median (IQR)	5 (0–10)	0 (0–5)	0.095	5 (0–14)	0 (0–5)	**0.047**
Cost of analgesic ($), median (IQR)	85.5 (66.8–104.4)	0.5 (0–18.9)	**< 0.001**	85.8 (66.8–117.7)	0.4 (0–18.9)	**< 0.001**
Cost of hospitalization ($), median (IQR)	3,778 (2,741–4,859)	2,273 (1,737–4,355)	**0.007**	3,723 (2,780–5,185)	2,273 (1,601–3,903)	**0.006**

IQR, interquartile range; HDU, highly dependency unit; ICU, intensive care unit; APFC, acute peripancreatic fluid collection; ANC, acute necrotic collection.

Statistical significance between groups (*p* ≤ 0.05) is indicated in bold.

The cumulative dosage of opioid consumption in the hydromorphone group (46.7 mg, IQR 31.5–67.2 mg) was higher than that in the pethidine group (5 mg, IQR 0–10 mg, *p* < 0.001) (Supplementary Table S2). The daily opioid consumption in both groups can be found in Supplementary Tables S3, 4. The dosage of dezocine as the rescue analgesia was similar and had no significant difference between the groups (*p* > 0.05). The cost of analgesics and hospitalization in the hydromorphone group were higher than those in the pethidine group (*p* < 0.05).

There were no significant differences in the daily Modified Marshall score and APACHE II score between the two groups (*p* > 0.05). The SOFA score on 48 h after admission in the hydromorphone group was higher than that in the pethidine group (*p* = 0.011). The BISAP scores on 48 and 72 h after admission in the hydromorphone group were higher than those in the pethidine group (0, IQR 0–1, *p* < 0.05), based on per-protocol analysis. The PASS scores in the hydromorphone group from day 1 to day 3 were significantly higher than those in the pethidine group (*p* < 0.05). Daily clinical scores are shown in Supplementary Table S5.

After treatment for three consecutive days, the serum levels of CRP, TNF-α, PCT, IL-6, IL-8 and IL-10 between the groups were not significantly different (*p* > 0.05) (Supplementary Table S6).

#### 3.4 Adverse events

A total of nine patients showed AEs, eight in the hydromorphone group and one in the pethidine group (*p* = 0.087) (Table 4). Specifically, five patients in the hydromorphone group and one in the pethidine group felt nausea and experienced vomiting during the use of medication. One male patient in the hydromorphone group showed urine retention on the first day after admission and dropped out (Parker et al., 1997; Goodarzi, 1999). After placing the urine catheter and discontinuing hydromorphone, urine retention disappeared. One male patient had a numbness sensation on his face and tongue within 24 h of hydromorphone PCA, which was relieved soon after the administration of intravenous 2 g calcium gluconate. One female suffered from 50 ml of bloody stool in the first hour after hydromorphone PCA, but the bleeding stopped after giving 2 units of hemocoagulase atrox.

**TABLE 4:** Adverse events in 77 acute pancreatitis patients.

Group	Hydromorphone (*n* = 39)	Pethidine (*n* = 38)	*p*-value
Adverse events, n (%)	8 (20.5)	1 (2.6)	0.087
Nausea/vomiting	5 (12.8)	1 (2.6)	
Urine retention	1 (2.6)	0	
Paresthesia	1 (2.6)	0	
Gastrointestinal bleeding	1 (2.6)	0	

## 4 Disscussion

To the best of our knowledge, this is the first RCT evaluating the safety and efficacy of intravenous hydromorphone PCA in AP patients. The strengths of this study include the following: 1) our treatment design the novel analgesic technology PCA versus the traditional analgesia in AP; 2) according to our inclusion criteria, 70% of patients were MSAP to SAP, compared to a high proportion of MAP cases in the existing 12 RCTs (Cai et al., 2021); 3) the onset of symptoms of patients in the two groups was limited within 72 h, which maintained homogeneity between the groups; and 4) a VAS score for pain intensity greater than five on admission was one of the inclusion criteria to more accurately evaluate the analgesic effects of the drugs, which was not clearly defined in most of the previous RCTs.

For the primary outcome, we did not find that intravenous hydromorphone PCA was superior to intramuscular pethidine in terms of analgesic effects, which was consistent with some previous studies comparing the two opioid drugs (Blamey et al., 1984; Stevens et al., 2002). In another study, epidural versus PCA opioids only had better analgesic effects on day 10 (Sadowski et al., 2015). Based on this result and the possibility that multiple factors likely interfere with analgesic effects in the late period of AP, we only evaluated analgesic effects based on VAS within the first 72 h in the two groups.

The interim analysis also found that hydromorphone PCA is associated with severity aggravation. Saluja and others (Barlass et al., 2018) have shown that morphine worsens AP severity and delays regeneration. Similar results were obtained in another experiment, whereby hydromorphone could aggravate the severity of AP models (Cheema et al., 2018). The results from basic experiments might explain, at least partly, our trial findings. A cohort study found in 1.14 million patients that the risk of opioid-related AEs increased with increasing opioid dosages (Herzig et al., 2014). However, the mean daily dosage of opioid consumption in our study was lower than 68 mg of oral morphine equivalents in that study. The cost of hydromorphone and PCA pump is more expensive than that of pethidine, indicating that hydromorphone PCA has a relatively poor health-economic value in AP pain management.

Hydromorphone PCA is also associated with a higher incidence of local complications. A recent study using AP models found that fentanyl pre-treatment exacerbated pancreatic necrosis and buprenorphine pre-treatment increased pancreatic edema (Bálint et al., 2022). These results were similar to our findings.

A potentially dangerous opioid-related AE is respiratory depression. However, we did not detect the occurrence of respiratory depression from patient self-reports, which might result from the low medication dose used in this study. However, over 20% of participants reported AEs during hydromorphone administration, which represents a higher incidence than that of the pethidine group. Nausea, vomiting, and urine retention—common side effects previously reported for opioid use (Benyamin et al., 2008) —occurred more frequently in the hydromorphone group. Gastrointestinal bleeding occurred in one patient in the hydromorphone group, and is a common side effect of NSAIDs but had not been reported during hydromorphone use (Lanas, 2012).

Our study has several limitations. First, researcher and participant blinding could not be performed, given the different administration of PCA and intramuscular injection. Bias during clinical observations and data collection by participants and researchers could not be absolutely eliminated, although outcome assessments and data analyses were conducted by an independent researcher blinded to the groups. Second, we used a relatively small sample size (77 participants). However, ours has been one of the largest sample size trials in AP pain management. Third, the control administration was designed as intramuscular pethidine based on the fact that there is no standard AP pain relief strategy and based on a recently published trial (Wilson et al., 2018).

In conclusion, intravenous hydromorphone PCA did not have superior analgesic effects or sufficient safety in comparison with intramuscular pethidine in AP pain management. We do not strongly recommend the application of hydromorphone PCA in the initial period of AP. Future multicenter randomized controlled trials with a large sample size should be conducted to validate the efficacy and safety of hydromorphone in patients with AP.

## Data Availability

The original contributions presented in the study are included in the article/Supplementary Materials, further inquiries can be directed to the corresponding authors.
