# Supplementary material for: Safety and efficacy of intravenous hydromorphone patient-controlled analgesia versus intramuscular pethidine in acute pancreatitis: An open-label, randomized controlled trial
Source: Front Pharmacol. 2022 Aug 4;13:962671. doi: 10.3389/fphar.2022.962671 (PMC9387897; doi:10.3389/fphar.2022.962671)
Supplement: Supplementary file 2 [file DataSheet1.docx]

Supplementary Material

# Supplementary Data

## Definition for Study Variables

Hypertriglyceridemia etiology was defined as admission serum triglyceride level ≥ 11.3 mmol/l or serum triglyceride level ≥ 5.65 mmol/l with chyle blood (Zhang et al., 2019). Biliary etiology was diagnosed as confirmation of gallstones using radiological imaging (including abdominal ultrasound, CT and MRI) (van Geenen et al., 2010). Alcohol etiology was diagnosed that an individual had four to five drinks daily over a period of more than 5 years (Coté et al., 2011; Forsmark et al., 2016).

Organ failure was identified using Sequential Organ Failure Assessment (SOFA) scoring by a score of ≥ 2 for individual organs (respiratory, cardiovascular, or renal systems). Transient organ failure was defined as organ failure that resolved within 48 hours. Persistent organ failure was defined as organ failure that lasted longer than 48 hours (occurring on 3 consecutive days) (Dellinger et al., 2012). Local complications were defined as in the revised Atlanta classification (Banks et al., 2013).

## Use of traditional Chinese medicine

In our department patients received a Chinese medicine herb formula called chaiqing chengqi decoction (CQCQCD) through either oral/nasogastric (50-200 ml) or enema (200 ml), with a frequency from once a day to every two hours at the doctor’s advice. The CQCQD is composed of 13 Chinese herbs which contain *Rhei Radix et Rhizoma* (Dahuang), *Magnoliae Officinalis Cortex* (Houpu), *Gardeniae Fructus* (Zhizi), *Bupleuri Radix* (Zhuyechaihu), *Scutellariae Radix* (Huangqin), *Chuanxiong Rhizoma* (Chuanxiong), *Carthami Flos* (Honghua), *Aurantii Fructus Immaturus* (Zhishi), *Corydalis Rhizoma* (Yanhusuo), *Auklandiae Radix* (Muxiang), *Paeonia Radix Rubra* (Chishao), *Glycyrrhizae Radix et Rhizoma* (Gancao) and *Natrii Sulfas* (Mangxiao).

# References

Zhang, R., Deng, L., Jin, T., Zhu, P., Shi, N., Jiang, K., et al. (2019). Hypertriglyceridaemia-associated acute pancreatitis: diagnosis and impact on severity. *HPB (Oxford)* 21(9)**,** 1240-1249. doi: 10.1016/j.hpb.2019.01.015.

van Geenen, E.J., van der Peet, D.L., Bhagirath, P., Mulder, C.J., and Bruno, M.J. (2010). Etiology and diagnosis of acute biliary pancreatitis. *Nat Rev Gastroenterol Hepatol* 7(9)**,** 495-502. doi: 10.1038/nrgastro.2010.114.

Coté, G.A., Yadav, D., Slivka, A., Hawes, R.H., Anderson, M.A., Burton, F.R., et al. (2011). Alcohol and smoking as risk factors in an epidemiology study of patients with chronic pancreatitis. *Clin Gastroenterol Hepatol* 9(3)**,** 266-273; quiz e227. doi: 10.1016/j.cgh.2010.10.015.

Forsmark, C.E., Vege, S.S., and Wilcox, C.M. (2016). Acute Pancreatitis. *N Engl J Med* 375(20)**,** 1972-1981. doi: 10.1056/NEJMra1505202.

Dellinger, E.P., Forsmark, C.E., Layer, P., Lévy, P., Maraví-Poma, E., Petrov, M.S., et al. (2012). Determinant-based classification of acute pancreatitis severity: an international multidisciplinary consultation. *Ann Surg* 256(6)**,** 875-880. doi: 10.1097/SLA.0b013e318256f778.

Banks, P.A., Bollen, T.L., Dervenis, C., Gooszen, H.G., Johnson, C.D., Sarr, M.G., et al. (2013). Classification of acute pancreatitis--2012: revision of the Atlanta classification and definitions by international consensus. *Gut* 62(1)**,** 102-111. doi: 10.1136/gutjnl-2012-302779.

# Supplementary Tables

# Supplementary Table 1. Cumulative VAS score based on intention-to-treat and per-protocol analyses.

| **Variables** | **Intention-to-treat analysis** | | | **Per-protocol analysis** | | |
| --- | --- | --- | --- | --- | --- | --- |
|  | **Hydromorphone**  **(*n* = 39)** | **Pethidine**  **(*n* = 38)** | ***p*-value** | **Hydromorphone**  **(*n* = 36)** | **Pethidine**  **(*n* = 36)** | ***p*-value** |
| Cumulative of VAS score, mean (s.d.) |  |  |  |  |  |  |
| 0-24 h | 22.59 (6.58) | 22.21 (7.84) | 0.819 | 23 (7) | 22 (8) | 0.686 |
| 0-48 h | 32.90 (11.74) | 33.18 (11.68) | 0.915 | 33 (12) | 34 (12) | 0.843 |
| 0-72 h | 41.82 (18.96) | 42.13 (17.93) | 0.941 | 41 (19) | 43 (18) | 0.850 |

VAS, visual analog scale score.

**Supplementary Table 2.** Consumption of opioids at different times based on intention-to-treat and per-protocol analyses.

| **Variables** | **Intention-to-treat analysis** | | | **Per-protocol analysis** | | |
| --- | --- | --- | --- | --- | --- | --- |
|  | **Hydromorphone**  **(*n* = 39)** | **Pethidine**  **(*n* = 38)** | ***p*-value** | **Hydromorphone**  **(*n* = 36)** | **Pethidine**  **(*n* = 36)** | ***p*-value** |
| Consumption of opioids (mg), median (IQR) |  |  |  |  |  |  |
| 0-24 h | 18.3 (11.9-30.5) | 5 (0-5) | **< 0.001** | 18.9 (11.9-31.85) | 5 (0-5) | **< 0.001** |
| 24-48 h | 13.4 (8.4-19.6) | 0 (0-0) | **< 0.001** | 15.1 (8.4-15.1) | 0 (0-0) | **< 0.001** |
| 48-72 h | 9.8 (8.4-19) | 0 (0-0) | **< 0.001** | 10.15 (8.5-20.1) | 0 (0-0) | **< 0.001** |
| < 72 h | 46.7 (31.5-67.2) | 5 (0-10) | **< 0.001** | 50.3 (33.2-68.5) | 5 (0-10) | **< 0.001** |
| > 72 h | 5 (0-15) | 0 (0-5) | **0.030** | 7.5 (0-15) | 0 (0-5) | **0.007** |
| Total | 55.6 (32.7-77.2) | 5 (0-15) | **< 0.001** | 57.48 (36.6-80.8) | 5 (0-15) | **< 0.001** |

IQR, interquartile range.

**Supplementary Table 3.** The use of intravenous hydromorphone PCA in 39 acute pancreatitis patients.

| **Variables** | Values |
| --- | --- |
| PCA pump press numbers, median (IQR) |  |
| 0-24 h | 6 (2-11) |
| 24-48 h | 2 (0-8) |
| 48-72 h | 1 (0-5) |
| total | 14 (3-29) |
| Consumption of hydromorphone (mg), median (IQR) |  |
| 0-24 h | 2.5 (1.5-3.5) |
| 24-48 h | 1.6 (1.2-2.8) |
| 48-72 h | 1.4 (1.2-2.2) |
| total | 6.3 (4.0-9.5) |

IQR, interquartile range; PCA, patient-controlled analgesia.

**Supplementary Table 4.** The use of pethidine in 38 acute pancreatitis patients.

| **Variables** | Values |
| --- | --- |
| Number of patients request pethidine, median (IQR) |  |
| 0-24 h | 1 (0-1.3) |
| 24-48 h | 0 (0-0) |
| 48-72 h | 0 (0-0) |
| total | 1 (0-2) |
| Consumption of pethidine (mg), median (IQR) |  |
| 0-24 h | 5 (0-5) |
| 24-48 h | 0 (0-0) |
| 48-72 h | 0 (0-0) |
| total | 5 (0-10) |

IQR, interquartile range.

**Supplementary Table 5.** Daily evaluation of clinical score systems based on intention-to-treat and per-protocol analyses.

| **Variables** | **Intention-to-treat analysis** | | | **Per-protocol analysis** | | |
| --- | --- | --- | --- | --- | --- | --- |
|  | **Hydromorphone**  **(*n* = 39)** | **Pethidine**  **(*n* = 38)** | ***p*-value** | **Hydromorphone**  **(*n* = 36)** | **Pethidine**  **(*n* = 36)** | ***p*-value** |
| Modified Marshal, median (IQR) |  |  |  |  |  |  |
| 24 h | 2 (0-2) | 1 (0-2) | 0.401 | 1 (0-2) | 1 (0-2) | 0.440 |
| 48 h | 1 (0-2) | 0 (0-1) | 0.093 | 1 (0-2) | 0 (0-1) | 0.066 |
| 72 h | 1 (0-2) | 0 (0-1) | 0.207 | 0 (0-2) | 0 (0-1) | 0.161 |
| SOFA, median (IQR) |  |  |  |  |  |  |
| 24 h | 3 (1-4) | 2 (1-4) | 0.378 | 2 (1-4) | 2 (1-3) | 0.309 |
| 48 h | 2 (1-4) | 1 (0-3) | **0.011** | 2 (1-4) | 1 (0-3) | **0.008** |
| 72 h | 2 (1-4) | 2 (0-3) | 0.302 | 2 (0-4) | 1.5 (0-3) | 0.191 |
| BISAP, median (IQR) |  |  |  |  |  |  |
| 24 h | 1 (0-2) | 1 (0-1) | 0.770 | 1 (0-2) | 1 (0-1) | 0.596 |
| 48 h | 1 (1-2) | 0 (0-1) | **0.027** | 1 (1-2) | 0 (0-1) | **0.016** |
| 72 h | 1 (0-1) | 0 (0-1) | 0.061 | 1 (0-1) | 0 (0-1) | **0.047** |
| APACHE II, median (IQR) |  |  |  |  |  |  |
| 24 h | 6 (4-10) | 5.5 (4-8) | 0.499 | 5.5 (4-10) | 5 (4-7) | 0.386 |
| 48 h | 5 (4-8) | 4 (2.5-6) | 0.093 | 5 (3-8) | 4 (2-6) | 0.052 |
| 72 h | 4 (2-7) | 4 (2-6) | 0.259 | 4 (2-7) | 2 (2-3) | 0.209 |
| PASS, median (IQR) |  |  |  |  |  |  |
| 24 h | 253 (187-320) | 183 (125-245) | **0.001** | 258 (193-319) | 170 (125-239) | **< 0.001** |
| 48 h | 222 (139-278) | 85 (55-173) | **< 0.001** | 223 (146-278) | 83 (55-151) | **< 0.001** |
| 72 h | 165 (100-247) | 65 (48-110) | **< 0.001** | 183 (102-249) | 60 (46-109) | **< 0.001** |

IQR, interquartile range; SOFA, sequential organ failure assessment; BISAP, bedside index of severity in acute pancreatitis; APACHE II, acute physiology and chronic health evaluation; PASS, pancreatitis activity scoring system.

**Supplementary Table 6.** Serum levels of CRP, TNF-α, PCT, IL-6, IL-8 and IL-10 at different time based on intention-to-treat and per-protocol analyses.

| **Parameters** | **Intention-to-treat analysis** | | | **Per-protocol analysis** | | |
| --- | --- | --- | --- | --- | --- | --- |
| **Group** | **Hydromorphone**  **(*n* = 39)** | **Pethidine**  **(*n* = 38)** | ***p*-value** | **Hydromorphone**  **(*n* = 36)** | **Pethidine**  **(*n* = 36)** | ***p*-value** |
| CRP (mg/L) |  |  |  |  |  |  |
| Admission, median (IQR) | 171.5 (22.9-317.8) | 117.5 (12.0-248.8) | 0.256 | 171 (24.6-314) | 121.5 (15.7-253.5) | 0.368 |
| Day 4, mean (s.d.) | 191.5 (116.7) | 162.8 (109.9) | 0.741 | 189.8 (116.3) | 164.4 (111.1) | 0.353 |
| TNF-α (pg/mL), median (IQR) |  |  |  |  |  |  |
| Admission | 8.0 (6.2-10.2) | 7.1 (5.5-9.8) | 0.428 | 8.0 (6.1-9.9) | 6.8 (5.4-9.7) | 0.379 |
| Day 4 | 8.7 (6.1-13.1) | 8.2 (6.6-9.6) | 0.302 | 8.4 (6.4-11.3) | 8.2 (6.6-9.6) | 0.369 |
| PCT (ng/mL), median (IQR) |  |  |  |  |  |  |
| Admission | 0.6 (0.1-1.5) | 0.2 (0.1-1.7) | 0.317 | 0.6 (0.1-1.5) | 0.2 (0.1-1.8) | 0.310 |
| Day 4 | 0.6 (0.2-1.1) | 0.3 (0.1-1.0) | 0.214 | 0.6 (0.3-1.1) | 0.3 (0.1-1.0) | 0.163 |
| IL-6 (pg/mL), median (IQR) |  |  |  |  |  |  |
| Admission | 268 (66.1-397.0) | 118.9 (46.3-278.5) | 0.107 | 276 (80.5-430.1) | 104.9 (45.6-270.8) | 0.041 |
| Day 4 | 46.5 (15.1-94.9) | 41.5 (10.3-76.9) | 0.265 | 46.3 (15.6-102.1) | 42.2 (9.7-77.6) | 0.277 |
| IL-8 (pg/mL), median (IQR) |  |  |  |  |  |  |
| Admission | 21.3 (11.6-55.8) | 31.5 (12.1-56.4) | 0.833 | 22.2 (11.9-58.7) | 22.7 (11.2-49.8) | 0.856 |
| Day 4 | 18.8 (12.2-37.9) | 17 (9.8-42.4) | 0.936 | 18.6 (9.6-28.4) | 17 (9.8-42.4) | 0.888 |
| IL-10 (pg/mL), median (IQR) |  |  |  |  |  |  |
| Admission | 8.3 (5-13.8) | 6.3 (5-10.7) | 0.341 | 8.94 (5-15.9) | 6.2 (5-9.7) | 0.104 |
| Day 4 | 5 (5-5) | 5 (5-5) | 0.527 | 5 (5-5) | 5 (5-5) | 0.426 |

IQR, interquartile range; CRP, C-reactive protein; TNF, tumor necrosis factor; PCT, procalcitonin; IL, interleukin.
